# Comparative analysis of mortality in patients admitted with an infection with influenza A/B virus, respiratory syncytial virus, rhinovirus, metapneumovirus or SARS‐CoV‐2

**DOI:** 10.1111/irv.13237

**Published:** 2024-01-18

**Authors:** Hanneke Boon, Arend‐Jan Meinders, Erik Jan van Hannen, Matthijs Tersmette, Erik Schaftenaar

**Affiliations:** ^1^ Department of Medical Microbiology and Immunology St. Antonius Hospital the Netherlands; ^2^ Department of Internal Medicine St. Antonius Hospital the Netherlands; ^3^ Intensive Care Unit St. Antonius Hospital the Netherlands

**Keywords:** influenza, metapneumovirus, mortality, respiratory syncytial virus, rhinovirus, SARS‐CoV‐2

## Abstract

**Background:**

While influenza virus and severe acute respiratory syndrome coronavirus 2 (SARS‐CoV‐2) are recognised as a cause of severe illness and mortality, clinical interest for respiratory syncytial virus (RSV), rhinovirus and human metapneumovirus (hMPV) infections is still limited.

**Methods:**

We conducted a retrospective database study comparing baseline characteristics and 30‐day mortality in a large cohort of adult patients admitted for an overnight stay or longer with an influenza virus (A/B), rhinovirus, hMPV, RSV or SARS‐CoV‐2 infection. For non‐SARS‐CoV‐2 viruses, data were included for the period July 2017–February 2020. For SARS‐CoV‐2, data between March 2020 and March 2022 were included.

**Results:**

Covariate‐adjusted 30‐day mortality following RSV, hMPV or rhinovirus infections was substantial (crude mortality 8–10%) and comparable with mortality following hospitalisation with an influenza A virus infection. Mortality following a SARS‐CoV‐2 infection was consistently higher than for any other respiratory virus, at any point in time (crude mortality 14–25%). Odds of mortality for SARS‐CoV‐2 compared with influenza A declined from 4.9 to 1.7 over the course of the pandemic. Patients with SARS‐CoV‐2 infection had less comorbidity than patients with other respiratory virus infections and were more often male. In this cohort, age was related to mortality following hospitalisation, while an association with comorbidity was not apparent.

**Conclusions:**

With the exception of SARS‐CoV‐2 infections, we find the clinical outcome of common respiratory virus infections requiring hospitalisation more similar than often assumed. The observed mortality from SARS‐CoV‐2 was significantly higher, but the difference with other respiratory viruses became less distinct over time.

**Abbreviations**
CCI: Charlson Comorbidity Index
COVID‐19: coronavirus disease 2019
ICD: International Classification of Disease
hMPV: human metapneumovirus
NAAT: nucleic acid amplification test
(a)OR: (adjusted) odds ratio
RSV: respiratory syncytial virus
SARS‐CoV‐2: severe acute respiratory syndrome coronavirus 2

## 1 INTRODUCTION

Lower respiratory tract infections are among the five leading causes of morbidity and mortality worldwide.^1^ Prior to the coronavirus disease 2019 (COVID‐19) pandemic, respiratory viruses, in particular influenza and respiratory syncytial virus (RSV), were the main explanations for excess mortality in winter season.^2^, ^3^, ^4^ Non‐influenza respiratory viruses are less well‐studied, but may account for half of hospitalised respiratory infections during respiratory virus seasons.^5^

In March 2020, the focus of research on viral respiratory pathogens inevitably shifted towards severe acute respiratory syndrome coronavirus 2 (SARS‐CoV‐2). Early comparative analyses show higher mortality following hospitalisation with SARS‐CoV‐2 infection than following an influenza infection.^6^, ^7^ Mortality of hospitalised SARS‐CoV‐2 cases seems to have declined progressively over the course of the pandemic.^8^, ^9^, ^10^ However, the majority of studies published thus far only cover the first phases of the pandemic. Therefore, recent data on the later phases of the pandemic is needed for a more comprehensive perspective on mortality following a SARS‐CoV‐2 infection.

Upsurges of infections with common respiratory viruses have occurred in the wake of lessened public restrictions installed to mitigate the spread of SARS‐CoV‐2.^11^, ^12^ These upsurges have triggered concerns that SARS‐CoV‐2, influenza and other common respiratory viruses might co‐circulate in future, thereby further increasing pressure on healthcare in winter months.^13^ A more detailed understanding of patients' characteristics and outcomes of hospitalised viral respiratory infections is needed to plan and prepare for respiratory seasons to come.

Therefore, in this retrospective database study, clinical characteristics and 30‐day mortality are described for a large cohort of adult patients admitted with an influenza virus, rhinovirus, human metapneumovirus (hMPV), RSV or SARS‐CoV‐2 infection. For a more in‐depth comparison, trends in mortality following hospitalisation with a SARS‐CoV‐2 infection are examined over four consecutive half year periods between March 2020 and March 2022.

## 2 METHODS

### 2.1 Study design

A retrospective cohort study was conducted on all adult patients hospitalised with a laboratory confirmed viral respiratory infection between 1 July 2017 and 1 March 2022. The St. Antonius Hospital is a 500‐bed general teaching hospital in the Netherlands. Respiratory viruses of interest were influenza A virus, influenza B virus, RSV, rhinovirus, hMPV and SARS‐CoV‐2. Testing for influenza A/B virus, RSV, hMPV and rhinovirus was performed routinely in patients presenting with respiratory symptoms from July 2017 until end of February 2020, the start of the COVID‐19 pandemic. Two types of nucleic acid amplification (NAAT) tests were in use during the period of data collection, that is, reverse‐transcriptase PCR, where viral RNA was amplified after a reverse transcriptase step followed by PCR, and transcription mediated amplification (TMA). The hospital laboratory is ISO15189‐certified and in house‐laboratory tests have been developed and validated in accordance with ISO15189‐guidelines (see Table S1, ^14^, ^15^ for details on laboratory tests and manufacturers). To ensure unbiased, routine testing for non‐SARS‐CoV‐2 viruses, data inclusion on orders for these viruses was closed after 1 March 2020. SARS‐CoV‐2 was routinely tested in patients presenting with respiratory symptoms from March 2020 until the end of the study period. The local Medical Ethics Review Committee waived the necessity for formal approval of the study as well as the need for informed consent (reference AW22.020/W22.030).

### 2.2 Data sources and inclusion criteria

For data extraction, we first accessed the laboratory information system (GLIMS, CliniSys|MIPS, Belgium) to extract all positive NAAT results from respiratory specimen (throat and nasopharynx swabs, sputum or bronchoalveolar lavage) for the viruses under study (Table S1). Subjects who tested positive for more than one respiratory virus were excluded from the analyses. Detected viral co‐infections are presented in Table S2. The hospital's electronic health record database (Epic, Epic Systems Corporation, USA) was then queried to extract data on baseline clinical characteristics, medical history and mortality at 30 days for subjects with a positive NAAT result that were admitted to the hospital for an overnight stay or longer. Excluded were subjects whose samples had been collected more than 24 h before admission or 72 h after admission. In the case of more than one admission between 2017 and 2022 for the same virus, only the first episode for this patient was included. To describe evolution of SARS‐CoV‐2 mortality over time, independent of pandemic wave, changes in therapy, vaccination or circulating variant‐of‐concern, SARS‐CoV‐2 data was divided in equal six months' periods.

### 2.3 Study outcomes and definitions

The main outcome of this study was 30‐day mortality following hospital admission, regardless of hospital admission status at day 30. Data was collected on patients' basic characteristics (age at admission and sex), registered chronic comorbidities and survival at 30 days after hospital admission. Chronic comorbidities were recorded as International Classification of Disease (ICD‐) 10 codes and scored according to the updated Charlson Comorbidity Index (CCI).^16^

### 2.4 Statistical analysis

Continuous data are described as means (standard deviation, SD) if normally distributed and as median (interquartile range, IQR) if non‐normally distributed. Categorical data are summarized as number (n) and as proportion (%). Baseline characteristics of subjects and mortality are described per virus detected. For SARS‐CoV‐2 infections, data is described in four half year periods, based on date of hospital admission. Age and CCI were stratified based on quantiles of data. Missing values were assumed to be missing at random.

Crude differences in subjects' characteristics between virus groups or the different phases of the pandemic were investigated using chi‐square (χ^2^) test for nominal and Kruskal–Wallis tests for continuous variables. Mortality in the first 30 days following admission is described graphically using cumulative incidence plots. As death dates in the electronic health records have been validated against the Dutch Death Register, mortality status at the 30‐day mark is certain and no subjects were lost to follow‐up. Sex‐ and age‐adjusted logistic regression analyses were performed to compare over/underrepresentation of specific comorbidities in patients with a SARS‐CoV‐2 or influenza A virus infection.

Mortality at 30 days following hospital admission was modelled using both univariable and multivariable logistic regression analysis. Covariates in the multivariable regression models were selected a priori and included age, sex and score on the updated CCI. In the main model, all subjects were included and influenza A was chosen as the reference group (Table 2). Additional logistic regression models were constructed where 1) only SARS‐CoV‐2 data was included to compare mortality in the last recorded phase to the initial phase of the pandemic (see supplemental Table S3) or 2) only data on other common respiratory viruses was included to compare mortality between influenza A, B, RSV, hMPV and rhinovirus infections only (Table S3).

Statistical analyses were performed in RStudio (version 4.1.2.) using the ‘comorbidity’, ‘survminer’ and ‘glm’ packages.^17^ A *p*‐value <0.05 was considered statistically significant.

## 3 RESULTS

**FIGURE 1:**
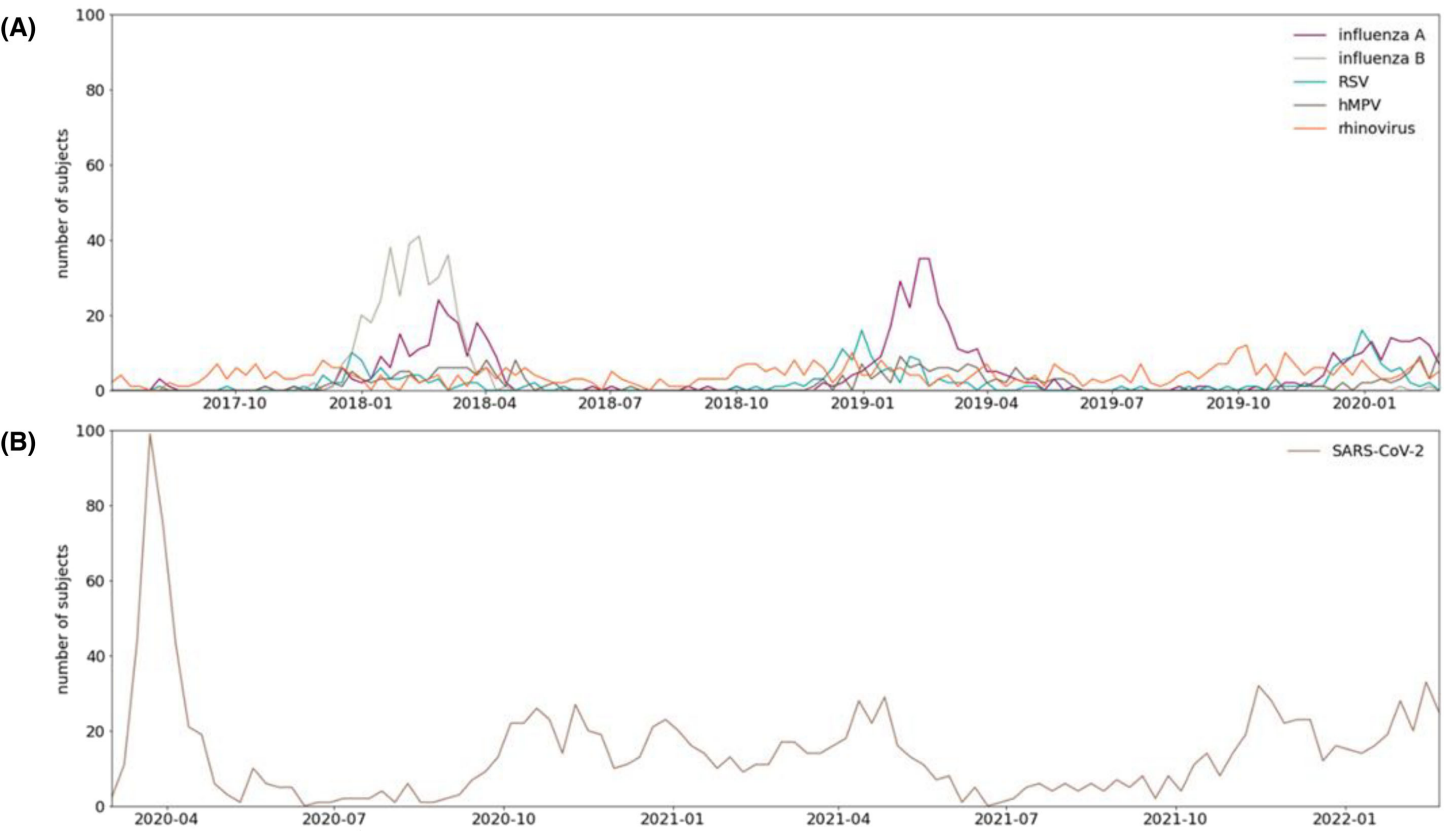
Number of patients admitted for at least an overnight stay between (A) 1 July 2017 and 1 March 2020 and (B) 1 March 2020 and 1 March 2022, following a positive PCR for influenza virus (A or B), RSV, rhinovirus, hMPV or SARS‐CoV‐2.

Included in the analysis were 3.504 adult patients with a NAAT‐confirmed infection of any of the respiratory viruses under study and admitted for more than an overnight stay (Table 1 and Figure 1A, B).

**TABLE 1:** Baseline characteristics.

			RSV			*p*‐Value other viruses^a^	SARS‐CoV‐2	SARS‐CoV‐2	SARS‐CoV‐2	SARS‐CoV‐2	*p*‐Value SARS‐CoV‐2^b^	*p*‐Value all viruses^c^	
	Influenza A	Influenza B	July 2017–Feb 2020	hMPV	Rhinovirus		March–Aug 2020	Sept 2020–Feb 2021	March–Aug 2021	Sept 2021–Feb 2022			
**Number of subjects with positive NAAT (n)**	919	517	365	374	922	<0.001	441	546	372	651	<0.001	<0.001	
**Number of subjects admitted (n, % of positive)**	613 (67 %)	364 (70 %)	260 (71 %)	251 (67 %)	550 (60 %)	<0.001	371 (84 %)	389 (71 %)	274 (74 %)	432 (66 %)	<0.001	<0.001	
**Baseline characteristics of admitted subjects**													
Male sex (%)	284 (46 %)	158 (43 %)	117 (45 %)	105 (42 %)	264 (48 %)	ns	229 (62 %)	218 (56 %)	154 (56 %)	247 (57 %)	ns	<0.001	
Age (year, median [IQR])	70 (60–80)	74 (66–81)	74 (65–83)	74 (64–84)	69 (58–78)	<0.001	66 (54–77)	73 (61–81)	65 (53–74)	71 (57–80)	<0.001	<0.001	
Age per category (n. %)						<0.001					<0.001	<0.001	
18–49	80 (13 %)	25 (7 %)	11 (4 %)	20 (8 %)	83 (15 %)		70 (19 %)	34 (9 %)	54 (20 %)	78 (18 %)			
50–59	69 (11 %)	29 (8 %)	35 (13 %)	24 (10 %)	69 (13 %)		70 (19 %)	53 (14 %)	59 (22 %)	47 (11 %)			
60–69	141 (23 %)	74 (20 %)	49 (19 %)	55 (22 %)	141 (26 %)		74 (20 %)	76 (20 %)	63 (23 %)	82 (19 %)			
69–80	180 (29 %)	127 (35 %)	77 (30 %)	70 (28 %)	140 (25 %)		103 (28 %)	122 (31 %)	64 (23 %)	115 (27 %)			
80 +	143 (23 %)	109 (30 %)	88 (34 %)	82 (33 %)	117 (21 %)		54 (15 %)	104 (27 %)	34 (12 %)	110 (25 %)			
CCI (points. Median (IQR)^d^	1 (1–3)	2 (1–3)	2 (1–4)	2 (1–3)	2 (1–3)	ns	0 (0–2)	1 (0–2)	0 (1–2)	1 (0–2)	0.04	<0.001	
CCI per category (n. %)^d^						<0.01					<0.001	<0.001	
0	148 (24 %)	73 (20 %)	44 (17 %)	42 (17 %)	93 (17 %)		214 (58 %)	166 (43 %)	137 (50 %)	190 (44 %)			
1 or 2	267 (44 %)	148 (41 %)	96 (37 %)	96 (38 %)	248 (45 %)		103 (28 %)	131 (34 %)	85 (31 %)	140 (32 %)			
3 or 4	129 (21 %)	90 (25 %)	77 (30 %)	71 (28 %)	132 (24 %)		35 (9 %)	54 (14 %)	41 (15 %)	64 (15 %)			
5 or more	68 (11 %)	53 (15 %)	41 (16 %)	42 (17 %)	77 (14 %)		19 (5 %)	37 (10 %)	8 (3 %)	35 (8 %)			
**Baseline characteristics**													
Comorbidity (n, %)												
Congestive heart failure	120 (20 %)	97 (27 %)	79 (30 %)	83 (33 %)	128 (23 %)	<0.001	31 (8 %)	60 (15 %)	29 (11 %)	74 (17 %)	<0. 001	<0.001
Chronic pulmonary disease	316 (52 %)	174 (48 %)	155 (60 %)	135 (54 %)	337 (61 %)	<0.001	67 (18 %)	95 (24 %)	60 (22 %)	108 (25 %)	0.08	<0.001
Diabetes with complications	28 (5 %)	19 (5 %)	13 (5 %)	21 (8 %)	24 (4 %)	ns	18 (5 %)	34 (9 %)	12 (4 %)	19 (4 %)	0.02	ns
Renal disease	63 (10 %)	64 (18 %)	39 (15 %)	37 (15 %)	63 (12 %)	<0.01	21 (6 %)	41 (11 %)	24 (9 %)	58 (13 %)	<0.01	<0.001
Any malignancy	136 (22 %)	105 (29 %)	67 (26 %)	66 (26 %)	143 (26 %)	ns	46 (12 %)	73 (19 %)	48 (18 %)	78 (18 %)	0.07	<0.001
Metastatic solid tumor	30 (5 %)	24 (7 %)	21 (8 %)	19 (8 %)	38 (7 %)	ns	8 (2 %)	24 (6 %)	6 (2 %)	18 (4 %)	<0.05	<0.001
**Outcome**												
30‐day mortality	53 (9 %)	38 (10 %)	27 (10 %)	23 (9 %)	45 (8 %)	ns	92 (25 %)	81 (21 %)	37 (14 %)	59 (14 %)	<0.001	<0.001

^a^ Comparison of influenza (A and B), RSV, hMPV and rhinovirus using χ^2^ test for nominal and Kruskal–Wallis test for continuous variables.

^b^ Comparison of phases of SARS‐CoV‐2 pandemic using χ^2^ test for nominal and Kruskal–Wallis test for continuous variables.

^c^ Comparison of influenza (A and B), RSV, hMPV, rhinovirus and SARS‐CoV‐2 (as phases) using χ^2^ test for nominal and Kruskal–Wallis test for continuous variables.

^d^ Based on ICD‐10 codes in medical history according to the updated CCI by Quan et al.^16^

### 3.1 Baseline characteristics

A peak in incidence during the winter season was observed for infection with influenza A/B and RSV. No season‐dependent patterns could be distinguished for the other viruses (Figure 1). Proportion of those admitted for more than an overnight stay following presentation to the hospital was lowest for rhinovirus infections (60%) but comparable for influenza, RSV or hMPV infections (67–71%, Table 1). Patients with a SARS‐CoV‐2 infection were younger than patients with an RSV, hMPV, influenza A or influenza B infection. Age of those with hMPV, RSV and influenza B infections was comparable. Patients with a SARS‐CoV‐2 infection were more often male than patients with any other viral respiratory infection (58% male, Table 1). Among patients with RSV, hMPV, rhinovirus, influenza A or influenza B infections, sex distribution was comparable. Patients with a SARS‐CoV‐2 infection had a lower CCI than those with any other respiratory infection (Table 1). In addition, patients with influenza A infections had lower CCI than those with hMPV, rhinovirus or RSV infections. Adjusted for age and sex, patients with SARS‐CoV‐2 less often had chronic heart failure, chronic pulmonary disease or malignancy than patients with other viral respiratory infections. Patients with influenza A less often had chronic heart failure than patients with RSV, hMPV or influenza B infections.

### 3.2 Differences in characteristics between patients in different phases of the SARS‐CoV‐2 pandemic

Proportion of those admitted for more than an overnight stay following presentation to the hospital was highest in the first phase of the pandemic (84%, Table 1). Over the course of the pandemic, patients with SARS‐CoV‐2 were more often male than female. Age and proportion with no comorbidities fluctuated over time (Table 1). Proportion of those with no comorbidities was lowest in March 2020–August 2020, in which 58% of those admitted had no comorbidities.

### 3.3 Crude 30‐day mortality

**FIGURE 2:**
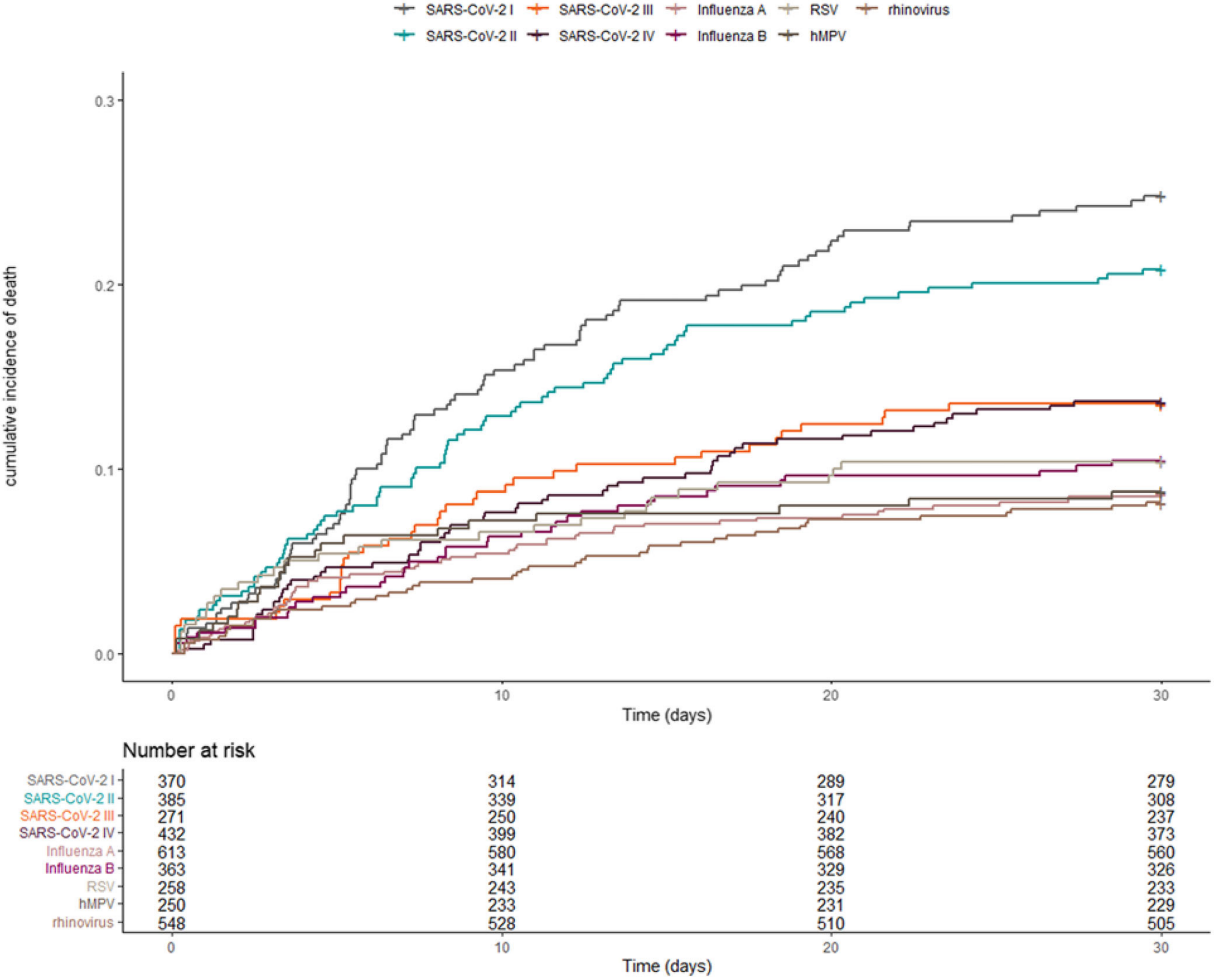
Cumulative incidence of death per virus detected, including number at risk from day of admission. Mortality of SARS‐CoV‐2 is divided in four phases of the pandemic, as described in the Methods section: (I) March 2020–August 2020, (II) September 2020–February 2021, (III) March 2021–August 2021 and (IV) September 2021–February 2022.

Crude mortality at 30 days following admission accumulated to 18% for SARS‐CoV‐2 infections, 9% for influenza A, 11% for influenza B, 10% for RSV, 8% for rhinovirus and 9% for hMPV infections (Table 1, Figure 2). Crude SARS‐CoV‐2 mortality was higher than for all other viruses, though a progressive decline in mortality over the course of the pandemic was found (March–Aug 2020: 25%, Sept 2020–Feb 2021: 21%, March–Aug 2021: 14%, Sept 2021–Feb 2022: 14%). Crude mortality for all non‐SARS‐CoV‐2 viruses was comparable (Table 1, Figure 2).

### 3.4 Covariate‐adjusted 30‐day mortality

Following adjustment for covariates, 30‐day mortality odds ratio (OR) for SARS‐CoV‐2 infection, regardless of pandemic phase, was 2.70 (95% CI: 1.98–3.77) versus influenza A. No differences were found in adjusted ORs for 30‐day mortality of influenza B, RSV, rhinovirus and hMPV infections (aOR influenza B: 1.05 (95% CI: 0.67–1.64), aOR RSV: 1.05 (95% CI: 0.63–1.71), aOR hMPV: 0.93 (0.54–1.55) and aOR rhinovirus: 1.00 (95% CI: 0.65–1.52, influenza A as reference, Table 2). In this cohort, age was related to mortality following hospitalisation, while an association with comorbidity was not apparent.

**TABLE 2:** Univariable associations and multivariable logistic regression model for 30‐day mortality of all viruses.

	Univariable model		Multivariable model	
	Crude OR	*p*‐Value	Adjusted OR	*p*‐Value
**Male sex**	1.34 (1.13–1.68)	<0.01	1.20 (0.97–1.48)	ns
**Age (year)**				
18–59	ref	‐	ref	‐
60–69	3.10 (2.01–4.91)	< 0.001	3.60 (2.31–5.74)	< 0.001
70–79	5.89 (3.98–9.01)	< 0.001	6.83 (4.56–10.59)	< 0.001
80 +	9.11 (6.19–13.90)	< 0.001	12.00 (7.99–18.62)	< 0.001
**CCI per category**				
0	ref	‐	ref	‐
1 or 2	1.01 (0.80–1.29)	ns	1.14 (0.88–1.49)	ns
3 or 4	1.13 (0.86–1.50)	ns	1.07 (0.79–1.46)	ns
5 or more	1.08 (0.79–1.51)	ns	1.02 (0.70–1.47)	ns
**Virus**				
Influenza A	ref	‐	ref	‐
Influenza B	1.23 (0.79–1.90)	ns	1.05 (0.67–1.64)	ns
RSV	1.22 (0.74–1.98)	ns	1.05 (0.63–1.71)	ns
hMPV	1.07 (0.63–1.76)	ns	0.93 (0.54–1.55)	ns
rhinovirus	0.94 (0.62–1.42)	ns	1.00 (0.65–1.52)	ns
SARS‐CoV‐2 (March 2020 – Aug 2020)	3.48 (2.42–5.06)	< 0.001	4.92 (3.33–7.33)	< 0.001
SARS‐CoV‐2 (Sept 2020 – Feb 2021)	2.78 (1.92–4.05)	< 0.001	2.75 (1.87–4.07)	< 0.001
SARS‐CoV‐2 (March 2021 – Aug 2021)	1.65 (1.05–2.57)	< 0.05	2.31 (1.43–3.68)	< 0.001
SARS‐CoV‐2 (Sept 2021 – Feb 2022)	1.67 (1.13–2.48)	< 0.05	1.72 (1.15–2.59)	< 0.01

### 3.5 Temporal trends in adjusted 30‐day mortality following SARS‐CoV‐2 infection

Covariate‐adjusted SARS‐CoV‐2 mortality declined progressively over the course of the pandemic (Table 2). Risk for mortality (as adjusted OR) was greatest between March 2020 and August 2020 and demonstrated a nearly three‐fold decline from the first phase to the last phase on which data was available (aOR for March 2020–Aug 2020: 4.92 (95% CI: 3.33–7.33), aOR for Sept 2021–February 2022: 1.72 (95% CI: 1.15–2.59, versus influenza A, Table 2). Despite this decline, adjusted OR for mortality of SARS‐CoV‐2 infection versus influenza A remained consistently higher over the full course of the pandemic.

## 4 DISCUSSION

The aim of this study was to provide a comparative analysis of 30‐day mortality following hospitalisation with an infection with influenza A/B, RSV, rhinovirus, metapneumovirus or SARS‐CoV‐2. In this retrospective cohort study, we found that, once hospitalisation is required, covariate‐adjusted mortality for RSV, hMPV or rhinovirus infections was comparable and not different from mortality following hospitalisation with an influenza A/B virus infection. Throughout the year, RSV, rhinovirus and hMPV infections made up a substantial proportion of total respiratory virus infections requiring hospitalisation. RSV is increasingly recognised as a cause of severe illness and mortality in high‐risk older adults,^4^, ^5^, ^18^, ^19^, ^20^ while clinical interest for rhinovirus and hMPV infections is still limited. In our study, 30‐day mortality was not different between influenza and RSV, hMPV or rhinovirus infections (8–10%) and in line with previously published influenza and RSV data on adults.^4^, ^21^, ^22^ Lower mortality rates have been reported by some^6^, ^23^ and might be explained by a shorter follow‐up time when in‐hospital mortality rather than 30‐day mortality is used as an outcome, or by less stringent criteria for hospital admission. In line with the general consensus, we found that elderly are particularly vulnerable for poor outcomes of respiratory virus infections.^2^, ^20^, ^24^ In our dataset, however, comorbidity registered as CCI was not associated with mortality following hospitalisation with a respiratory virus infection. This may seem in contrast to other findings;^25^, ^26^, ^27^ however, this discrepancy might in part be explained by a mitigating effect of influenza vaccination in high‐risk individuals, or by a lower admission threshold for those with expected severe progression of disease.

Few studies have compared SARS‐CoV‐2 mortality during winter 2021/2022 with mortality following hospitalisation with several other common respiratory virus infections. In the last half year of the study period (1 September 2021 to 1 March 2022), odds of mortality following hospitalisation with a SARS‐CoV‐2 infection were 1.7 times greater than following an influenza virus infection. Our finding of a three‐fold decrease in SARS‐CoV‐2 mortality over the course of the pandemic thus far is consistent with other studies reporting declines in mortality rates following hospitalisation.^9^, ^10^, ^28^, ^29^, ^30^ In the initial phases of the pandemic, changes in admission decisions^31^ and improved supportive care^9^, ^32^, ^33^ are thought to have contributed most to the decline in mortality. Vaccination strategies gave a subsequent impulse to further decline of mortality risk as of 2021, though their effect may have been counteracted, at least briefly, by the rise of the more severe alpha variant.^32^, ^34^ Decreased intrinsic severity of the omicron virus variant, booster vaccination and an increasing proportion of the population with acquired immunity probably contributed further,^10^, ^29^, ^35^, ^36^, ^37^ though waning immunity and/or vaccine evasion remains a concern.^38^ Data from the later phases of the pandemic are scarce. Our current data suggest an ongoing decrease in mortality risk following hospitalisation, although at present, it is too early to definitively exclude resurges because of waning immunity and the spread of evasive and more virulent virus variants.

Our study has some limitations. No data is available on population prevalence or hospitalisation rate per virus under study, or any differences therein. Our study does not address burden or (severe) outcomes in cases where hospitalisation is not appropriate. Instead, our findings foremost serve to raise awareness among medical specialists that, once hospitalization is required, the virus detected (if not SARS‐CoV‐2) seems to be of minor importance in determining outcome of the acute respiratory infection.

Secondly, at the early peaks of the COVID‐19 pandemic, the decision to admit patients with a SARS‐CoV‐2 virus infection may have been influenced by the pressure on hospital beds and chances of survival. Registration or testing bias is also possible but expected to be limited, as all patients with respiratory symptoms were routinely tested for the viruses under study. As our dataset is a direct extraction from the laboratory information system, it by design contains all test results. Finally, it was beyond the scope of this retrospective database study to investigate causal relationships between patient characteristics and mortality. Thus, a mitigating effect of (SARS‐CoV‐2 and/or influenza) vaccination in high‐risk individuals cannot be excluded. However, these limitations do not affect our finding that, once severity of disease is such that hospitalisation is required, mortality following rhinovirus and hMPV infections is comparable with mortality following RSV infections, of which the latter is increasingly recognised as potentially severe.

In conclusion, our data imply that not only COVID‐19 and influenza, but also the other viral respiratory infections under study should be recognized as potentially severe and treated accordingly once hospitalisation is required. Greater attention for surveillance, development of vaccines and (antiviral) therapy options for respiratory viruses other than influenza and SARS‐CoV‐2 seems justified to reduce burden on both healthcare and the individual.

## DATA AVAILABILITY, DATA SOURCES AND INFORMED CONSENT

Data may be made available upon request but is subject to approval by the St Antonius Hospital. This publication does not contain material published previously in other sources. The local Medical Ethics Review Committee waived the necessity for formal approval of the study as well as the need for informed consent.

## AUTHOR CONTRIBUTIONS

**Hanneke Boon:** Conceptualization; data curation; formal analysis; methodology; visualization; writing—original draft. **Arend‐Jan Meinders:** Conceptualization; methodology; writing—review and editing. **Erik Jan van Hannen:** Data curation; visualization; writing—review and editing. **Matthijs Tersmette:** Conceptualization; methodology; supervision; writing—review and editing. **Erik Schaftenaar:** Conceptualization; methodology; supervision; writing—review and editing.

## CONFLICT OF INTEREST STATEMENT

The authors declare that they have no known competing financial interests or personal relationships that could have appeared to influence the work reported in this paper. No external funding was received to conduct this study.

### PEER REVIEW

The peer review history for this article is available at https://www.webofscience.com/api/gateway/wos/peer-review/10.1111/irv.13237.

## Supporting information


**Table S1.** Platforms and manufacturers of NAAT. LDT: Lab developed test. ^a^ Protocol from Erasmus MC (Rotterdam, the Netherlands), ^b^ unpublished PCR design, ^c^ [37], ^d^unpublished PCR design, ^e^ adapted from [38].
**Table S2.** Co‐infections excluded from analysis.
**Table S3.** Multivariable regression models for 30‐day mortality for SARS‐CoV‐2 per half year and for other common respiratory viruses.

## Data Availability

Data may be made available upon request but is subject to approval by the St Antonius Hospital. The local Medical Ethics Review Committee waived the necessity for formal approval of the study as well as the need for informed consent. This publication does not contain material published previously in other sources.
