# Supplementary material for: Comparative analysis of mortality in patients admitted with an infection with influenza A/B virus, respiratory syncytial virus, rhinovirus, metapneumovirus or SARS‐CoV‐2
Source: Influenza Other Respir Viruses. 2024 Jan 18;18(1):e13237. doi: 10.1111/irv.13237 (PMC10796251; doi:10.1111/irv.13237)
Supplement: Supplementary file 1 — Table S1. Platforms and manufacturers of NAAT. LDT: Lab developed test. a Protocol from Erasmus MC (Rotterdam, the Netherlands), b unpublished PCR design, c [37], dunpublished PCR design, e adapted from [38]. Table S2. Co‐infections excluded from analysis. Table S3. Multivariable regression models for 30‐day mortality for SARS‐CoV‐2 per half year and for other common respiratory viruses. [file IRV-18-e13237-s001.pptx]

## Slide 1
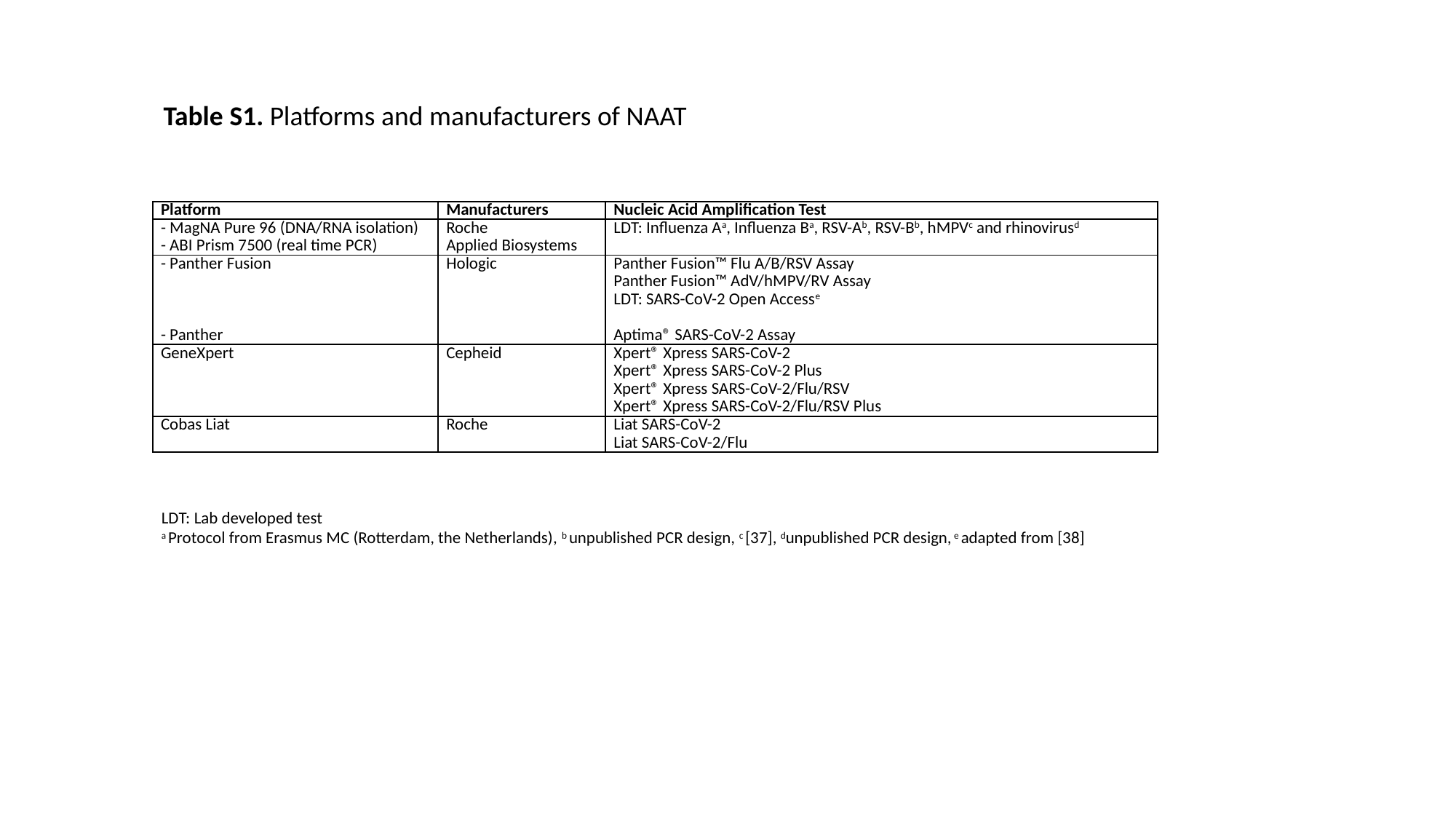

Table S1. Platforms and manufacturers of NAAT
| Platform | Manufacturers | Nucleic Acid Amplification Test |
| --- | --- | --- |
| - MagNA Pure 96 (DNA/RNA isolation) - ABI Prism 7500 (real time PCR) | Roche Applied Biosystems | LDT: Influenza Aa, Influenza Ba, RSV-Ab, RSV-Bb, hMPVc and rhinovirusd |
| - Panther Fusion       - Panther | Hologic | Panther Fusion™ Flu A/B/RSV Assay Panther Fusion™ AdV/hMPV/RV Assay LDT: SARS-CoV-2 Open Accesse   Aptima® SARS-CoV-2 Assay |
| GeneXpert | Cepheid | Xpert® Xpress SARS-CoV-2 Xpert® Xpress SARS-CoV-2 Plus Xpert® Xpress SARS-CoV-2/Flu/RSV Xpert® Xpress SARS-CoV-2/Flu/RSV Plus |
| Cobas Liat | Roche | Liat SARS-CoV-2 Liat SARS-CoV-2/Flu |
LDT: Lab developed test
a Protocol from Erasmus MC (Rotterdam, the Netherlands), b unpublished PCR design, c [37], dunpublished PCR design, e adapted from [38]

## Slide 2
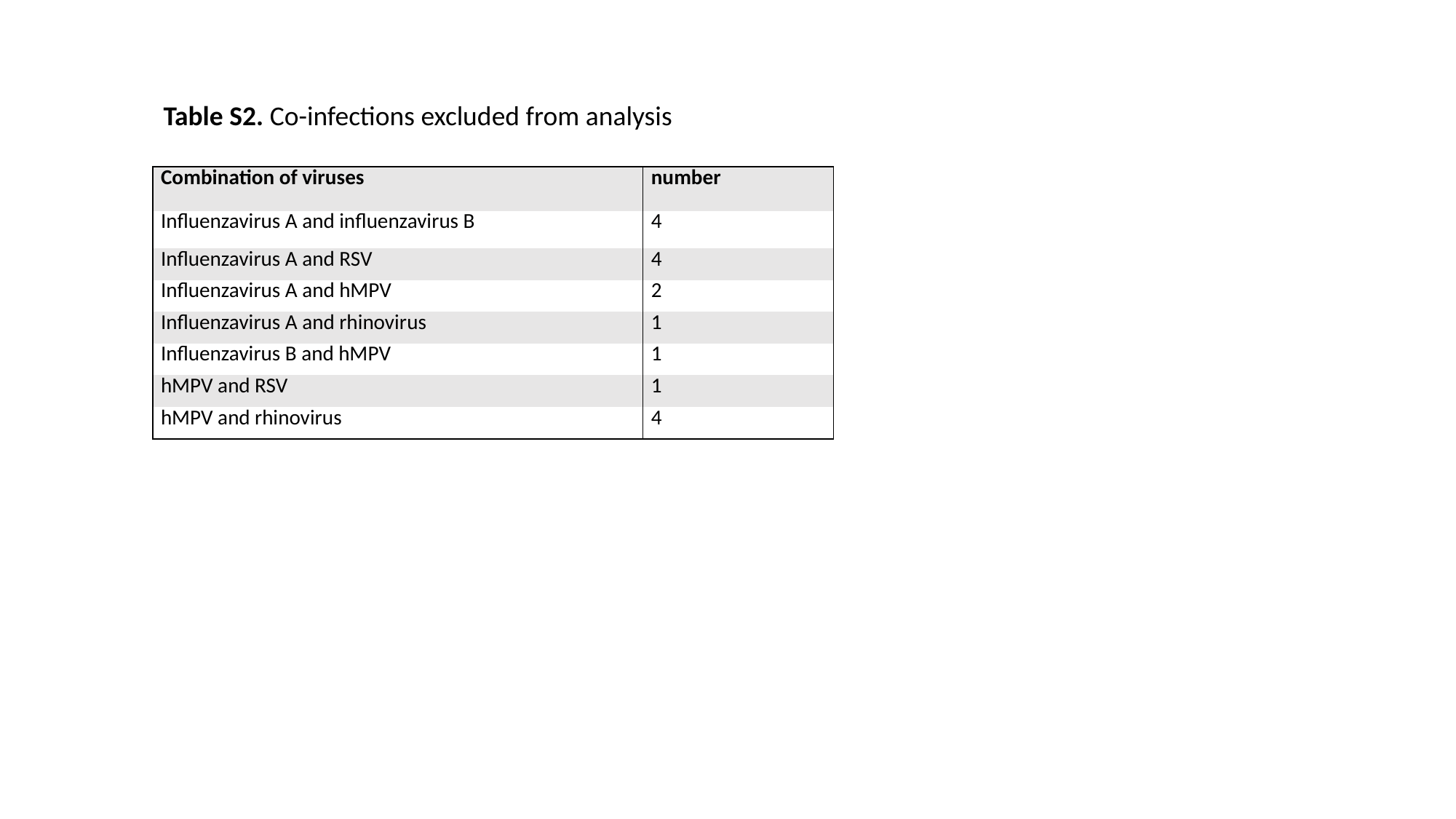

Table S2. Co-infections excluded from analysis
| Combination of viruses | number |
| --- | --- |
| Influenzavirus A and influenzavirus B | 4 |
| Influenzavirus A and RSV | 4 |
| Influenzavirus A and hMPV | 2 |
| Influenzavirus A and rhinovirus | 1 |
| Influenzavirus B and hMPV | 1 |
| hMPV and RSV | 1 |
| hMPV and rhinovirus | 4 |

## Slide 3
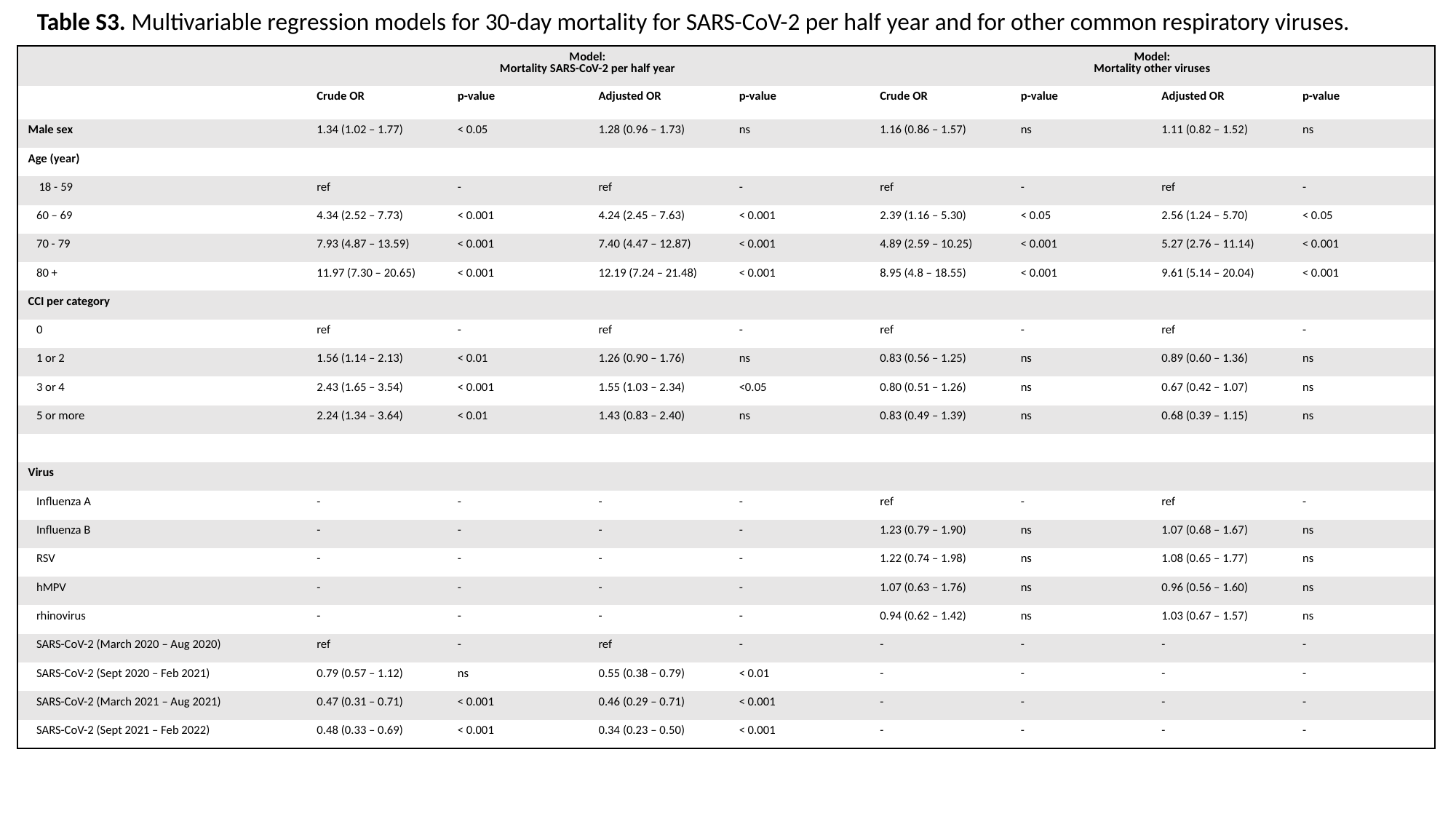

Table S3. Multivariable regression models for 30-day mortality for SARS-CoV-2 per half year and for other common respiratory viruses.
| | Model: Mortality SARS-CoV-2 per half year | | | | Model: Mortality other viruses | | | |
| --- | --- | --- | --- | --- | --- | --- | --- | --- |
| | Crude OR | p-value | Adjusted OR | p-value | Crude OR | p-value | Adjusted OR | p-value |
| Male sex | 1.34 (1.02 – 1.77) | < 0.05 | 1.28 (0.96 – 1.73) | ns | 1.16 (0.86 – 1.57) | ns | 1.11 (0.82 – 1.52) | ns |
| Age (year) | | | | | | | | |
| 18 - 59 | ref | - | ref | - | ref | - | ref | - |
| 60 – 69 | 4.34 (2.52 – 7.73) | < 0.001 | 4.24 (2.45 – 7.63) | < 0.001 | 2.39 (1.16 – 5.30) | < 0.05 | 2.56 (1.24 – 5.70) | < 0.05 |
| 70 - 79 | 7.93 (4.87 – 13.59) | < 0.001 | 7.40 (4.47 – 12.87) | < 0.001 | 4.89 (2.59 – 10.25) | < 0.001 | 5.27 (2.76 – 11.14) | < 0.001 |
| 80 + | 11.97 (7.30 – 20.65) | < 0.001 | 12.19 (7.24 – 21.48) | < 0.001 | 8.95 (4.8 – 18.55) | < 0.001 | 9.61 (5.14 – 20.04) | < 0.001 |
| CCI per category | | | | | | | | |
| 0 | ref | - | ref | - | ref | - | ref | - |
| 1 or 2 | 1.56 (1.14 – 2.13) | < 0.01 | 1.26 (0.90 – 1.76) | ns | 0.83 (0.56 – 1.25) | ns | 0.89 (0.60 – 1.36) | ns |
| 3 or 4 | 2.43 (1.65 – 3.54) | < 0.001 | 1.55 (1.03 – 2.34) | <0.05 | 0.80 (0.51 – 1.26) | ns | 0.67 (0.42 – 1.07) | ns |
| 5 or more | 2.24 (1.34 – 3.64) | < 0.01 | 1.43 (0.83 – 2.40) | ns | 0.83 (0.49 – 1.39) | ns | 0.68 (0.39 – 1.15) | ns |
| | | | | | | | | |
| Virus | | | | | | | | |
| Influenza A | - | - | - | - | ref | - | ref | - |
| Influenza B | - | - | - | - | 1.23 (0.79 – 1.90) | ns | 1.07 (0.68 – 1.67) | ns |
| RSV | - | - | - | - | 1.22 (0.74 – 1.98) | ns | 1.08 (0.65 – 1.77) | ns |
| hMPV | - | - | - | - | 1.07 (0.63 – 1.76) | ns | 0.96 (0.56 – 1.60) | ns |
| rhinovirus | - | - | - | - | 0.94 (0.62 – 1.42) | ns | 1.03 (0.67 – 1.57) | ns |
| SARS-CoV-2 (March 2020 – Aug 2020) | ref | - | ref | - | - | - | - | - |
| SARS-CoV-2 (Sept 2020 – Feb 2021) | 0.79 (0.57 – 1.12) | ns | 0.55 (0.38 – 0.79) | < 0.01 | - | - | - | - |
| SARS-CoV-2 (March 2021 – Aug 2021) | 0.47 (0.31 – 0.71) | < 0.001 | 0.46 (0.29 – 0.71) | < 0.001 | - | - | - | - |
| SARS-CoV-2 (Sept 2021 – Feb 2022) | 0.48 (0.33 – 0.69) | < 0.001 | 0.34 (0.23 – 0.50) | < 0.001 | - | - | - | - |
